# CsPAO2 Improves Salt Tolerance of Cucumber through the Interaction with CsPSA3 by Affecting Photosynthesis and Polyamine Conversion

**DOI:** 10.3390/ijms232012413

**Published:** 2022-10-17

**Authors:** Jianqiang Wu, Mengliang Zhu, Weikang Liu, Mohammad Shah Jahan, Qinsheng Gu, Sheng Shu, Jin Sun, Shirong Guo

**Affiliations:** 1 College of Horticulture, Nanjing Agricultural University, Nanjing 210095, China; 2 Department of Horticulture, Sher-e-Bangla Agricultural University, Dhaka 1207, Bangladesh; 3 Zhengzhou Fruit Research Institute, Chinese Academy of Agricultural Sciences, Zhengzhou 450009, China

**Keywords:** cucumber, salt stress, protein interaction, photosynthesis, polyamine

## Abstract

Polyamine oxidases (PAOs) are key enzymes in polyamine metabolism and are related to the tolerance of plants to abiotic stresses. In this study, overexpression of cucumber (*Cucumis sativus* L.) *PAO2* (*CsPAO2*) in Arabidopsis resulted in increased activity of the antioxidant enzyme and accelerated conversion from Put to Spd and Spm, while malondialdehyde content (MDA) and electrolyte leakage (EL) was decreased when compared with wild type, leading to enhanced plant growth under salt stress. Photosystem Ⅰ assembly 3 in cucumber (CsPSA3) was revealed as an interacting protein of CsPAO2 by screening yeast two-hybrid library combined with in vitro and in vivo methods. Then, *CsPAO2* and *CsPSA3* were silenced in cucumber via virus-mediated gene silencing (VIGS) with pV190 as the empty vector. Under salt stress, net photosynthetic rate (Pn) and transpiration rate (Tr) of *CsPAO2*-silencing plants were lower than pV190-silencing plants, and EL in root was higher than pV190-silencing plants, indicating that *CsPAO2*-silencing plants suffered more serious salt stress damage. However, photosynthetic parameters of *CsPSA3*-silencing plants were all higher than those of *CsPAO2* and pV190-silencing plants, thereby enhancing the photosynthesis process. Moreover, *CsPSA3* silencing reduced the EL in both leaves and roots when compared with *CsPAO2*-silencing plants, but the EL only in leaves was significantly lower than the other two gene-silencing plants, and conversion from Put to Spd and Spm in leaf was also promoted, suggesting that CsPSA3 interacts with CsPAO2 in leaves to participate in the regulation of salt tolerance through photosynthesis and polyamine conversion.

## 1. Introduction

Polyamines (PAs) are a kind of important physiologically bioactive substances in plants, which can indirectly affect the stress tolerance of plants through metabolism [1,2]. Polyamine oxidation is the main catabolic pathway of PAs, and polyamine oxidase (PAO) is responsible for catalyzing the oxidation of higher PAs whose amount of amino groups were more than two, such as Spermidine (Spd) or Spermine (Spm), which play a critical role in plant growth, development and stress responses. Kim et al. [3] found that AtPAO5 regulates Arabidopsis growth through thermospermine oxidase activity, and *AtPAO5* participates in auxin and cytokinin pathways to regulate xylem differentiation [4]. PAO is involved in the regulation of growth and development by light [5], whose levels are induced by light and associated with a light-inhibitory role in hypocotyl growth of maize. PAO can also regulate cell wall stiffening and cell growth rate through H_2_O_2_ produced by PAO function [6]. In addition, PAO is related to fruit development and ripening [7]. In one study, the Ca^2+^ channel of *AtPAO3* loss-of-function mutants were unable to open, which inhibited pollen tube growth and reduced the number of seeds [8].

Not only growth of root but also leaf is associated with PAO. Under salt stress, PAO induces cell elongation and helps maintain leaf growth in maize, thereby alleviating the inhibitory effect of stress on growth [9]. In Arabidopsis, genes encoding PAOs (*AtPAO2*-*4*) localized in peroxisome are induced by ABA, mechanical damage and salt stress [10,11]. The *PAO* expression level in *Salinity tolerance 1* (*St1*), a salt-tolerant wheat line, was found to be higher than the wild type, indicating that *PAO* has an important function in salt tolerance [12]. Pakdel et al. [13] compared two maize genotypes with different drought tolerance and found that *PAO* expression level, photosynthesis efficiency and antioxidant enzyme activities of relatively tolerant genotype (Karoon) was higher, suggesting that higher activity of polyamine oxidase and antioxidant enzymes might play a role in improving photosynthesis efficiency of this cultivar. Moreover, abiotic stress influenced plants’ responses to pathogen infection, the enhancement of PAO activity in grape induced by osmotic stress mediated expression of pathogen defense genes, which improved resistance to *Botrytis cinerea* [14]. Moreover, PAO function varies among different periods of growth and development. Gemes et al. [15] discovered that the effect of ZmPAO on salt tolerance of young tobacco plants was strong, while the effect became relatively moderate at the later stage of development. Overexpression of sweet orange *PAO4* in tobacco increased the seed germination rate but inhibited seedlings growth under salt stress [16].

Genes are transcribed and translated into functional proteins which play physiological roles. However, this process is not independent, and there is always an interaction between proteins. NADPH oxidase and PAO formed a feedback loop to regulate ROS homeostasis in tobacco, which affected stress tolerance [17]. Toumi et al. [18] found that under heat stress, silencing *HSP90* genes resulted in an elevated level of polyamine and acetylated polyamine, leading to enhanced *AtPAOs* expression, and proposed that HSP90s and PAO cross-talked to influence polyamine acetylation, oxidation and ROS homeostasis. To date, there have been few reports about the interacting proteins of PAO in plants.

Our previous study isolated and characterized PAO-encoding genes (*CsPAO1*-*4*) in cucumber [19], from which we could know that *CsPAO2*-*4* made obvious responses to NaCl treatment early in both root and leaf. It was found that there was a clear response to the treatment and the expression of *CsPAO2* was higher than the other member genes. Additionally, the *CsPAO2* expression shows apparent tissue-specificity with a much higher level in roots which is impacted primarily by salt. To further investigate the function of *CsPAOs* in responses to salt stress, in this study, as our knowledge, *CsPAO2* roles in salt stress were firstly investigated by heterogeneous expression of *CsPAO2* in Arabidopsis, and then screening of yeast two-hybrid (Y2H) cDNA library and virus-induced gene silencing (VIGS) was used to study the interacting functions of CsPAO2 protein and the molecular mechanism underlying the regulation of salt stress tolerance by them was explored.

## 2. Results

### 2.1. Biomass, Antioxidant Capacity and Polyamine Content of Arabidopsis Plants Overexpressing CsPAO2 under Salt Stress

**Figure 1:**
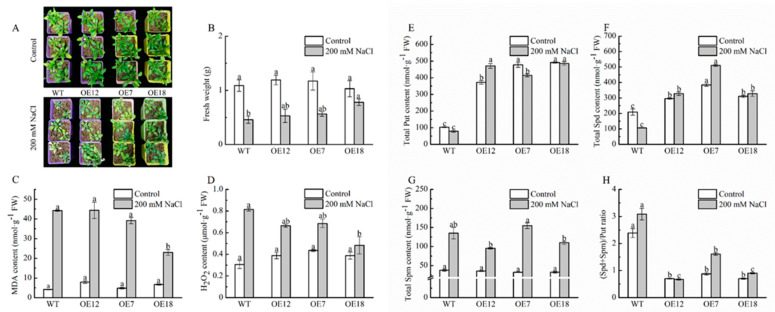
Plant growth (**A**,**B**) MDA, (**C**) H_2_O_2_ content, (**D**) polyamine content, (**E**–**H**) in leaf of wild type (WT) and transgenic Arabidopsis lines (OE7, OE12 and OE18). (**E**) Put; (**F**) Spd; (**G**) Spm; (**H**) (Spd+Spm)/Put ratio. Arabidopsis seedlings cultivated in vermiculite were irrigated with normal water or water containing 200 mM NaCl for 14 days. The different letters indicate significantly different test index values between different genotypes under the same treatment (*p* < 0.05) according to Tukey’s test.

Plant growth and antioxidant ability of WT and *CsPAO2*-overexpression Arabidopsis plants, whose expression levels of *CsPAO2* were ranked as follows: OE12, OE7 and OE18 (Figure S2), was investigated firstly. As shown in Figure 1A, growth of WT and transgenic lines was all inhibited after being treated with 200 mM NaCl, among which the plant size of overexpression plants was larger than that of WT and OE18 with the highest expression level of *CsPAO2* having more green leaves, leading to higher fresh weight in three transgenic lines, especially in OE18 (Figure 1B), whose fresh weight was remarkably higher than WT. This effect was more pronounced in plants that exposed to 300 mM NaCl stress (Figure S4D). In addition, compared with control plants, the MDA content in all salt-treated plants, whether in WT and transgenic lines, was increased greatly under salt stress. Notably, the MDA content of OE7 and OE18 was lower than that of WT, and there was significant difference between OE18 and other tested plants (Figure 1C). The same trend was also observed in H_2_O_2_ content (Figure 1D). Thus, oxidative damage caused by salt stress was alleviated in *CsPAO2*-overexpression Arabidopsis plants, which was associated with enhanced antioxidant capacity in overexpression lines, particularly POD and APX activity (Figure S5), contributing to improving salt stress tolerance and plant growth.

Considering that PAO is involved in polyamine metabolism, the polyamine content of WT and transgenic Arabidopsis plants was measured. As illustrated in Figure 1E,F, the Put and Spd contents were significantly higher in overexpression lines than those in WT under the control conditions, while the Spm content in three transgenic lines was reduced insignificantly, suggesting that CsPAO2 might catalyze the back-conversion of Spd or Spm, particularly Spm, which resulted in the obviously lower (Spd+Spm)/Put ratio in overexpression lines than WT (Figure 1H). After NaCl treatment, the Put contents of the treated plants except for OE12 were decreased (Figure 1E). Spd and Spm levels were raised in different ranges in transgenic plants with the largest range in OE7 whose ratio of (Spd+Spm)/Put increased about one fold under salt stress (Figure 1F–H), indicating that *CsPAO2* overexpression could promote Put’s conversion to Spd and Spm to keep the (Spd+Spm)/Put ratio from decreasing, and the declined increasing range in OE18 might be due to the higher expression level of *CsPAO2*.

### 2.2. CsPAO2 Interacts with PSA3

**Figure 2:**
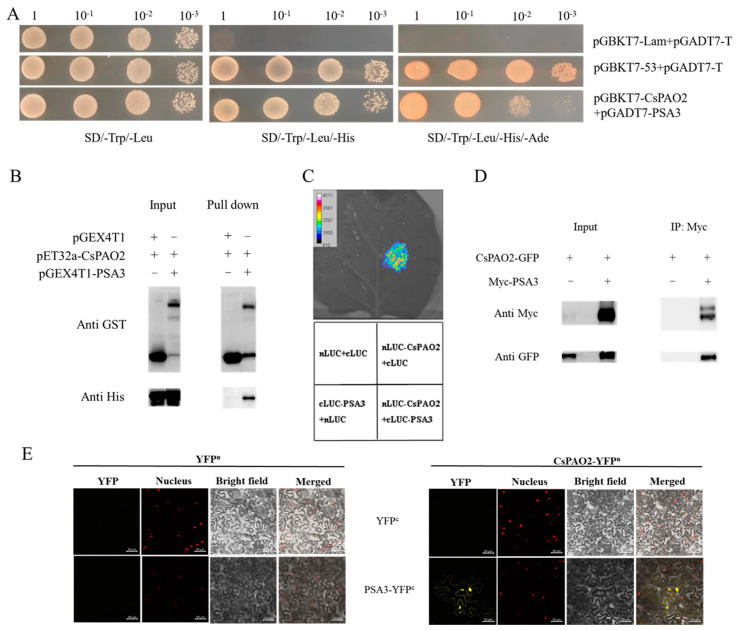
In vitro and in vivo validation of protein interaction between CsPAO2 and PSA3. (**A**) Yeast two-hybrid assay. CsPAO2 was ligated to DNA-binding domain (pGBKT7) as bait, while PSA3 was ligated to pGADT7. pGBKT7-Lam+pGADT7-T and pGBKT7-53+pGADT7-T was used as negative and positive control, respectively. (**B**) GST pull down assay. CsPAO2 and PSA3 was fused to pET32a and pGEX4T1, respectively. GST tag resin was used and proteins bound to the resin were detected by western blot with anti-GST and anti-His antibody. (**C**) LCA assay. CsPAO2 and PSA3 was ligated to nLUC and cLUC, respectively. Luciferase activity in tobacco leaf was determined at 48 h after agrobacteria infiltration. Empty vectors were transformed as control. (**D**) Co-IP assay. CsPAO2-GFP was expressed in tobacco leaves with or without Myc-PSA3, and Co-IP was performed by using Anti-c-Myc magnetic beads. Western blot was performed with anti-Myc and anti-GFP antibody. (**E**) BiFC assay. CsPAO2 was fused to N-terminal half of YFP (CsPAO2-YFP^n^), and PSA3 was fused to C-terminal half of YFP (PSA3-YFP^c^). Tobacco leaves co-transformed with empty YFP^n^ or YFP^c^ were used as control and observed using laser scanning confocal microscope. Scale bars (50 μm) were added to the bottom right of each figure using ZEN 2 software. Red signals represented nucleus localization.

To further study the roles of *CsPAO2* in salt stress responses, the yeast two-hybrid cDNA library was used to screen CsPAO2-interacting proteins, which were then verified by yeast two-hybrid assay. Unexpectedly, only the interaction between CsPAO2 and PSA3 was proven on SD/-Trp/-Leu/-His and SD/-Trp/-Leu/-His/-Ade plates (Figure 2A), and was testified by the following methods. Through GST pull down assay, a band was observed using anti-His antibody for proteins (pET32a-CsPAO2 and pGEX4T1-PSA3) bound with GST tag resin (Figure 2B), which supported the interaction of CsPAO2 with PSA3. Similarly, light was detected in the leaf region injected with nLUC-CsPAO2+cLUC-PSA3 (Figure 2C), indicating that there was interaction between this two proteins. Consistent with the results of the experiments mentioned above, Co-IP assay showed that CsPAO2 interacted with PSA3 in plants (Figure 2D). BiFC assay confirmed this positive interaction, as shown in Figure 2E, YFP fluorescent signals were observed in tobacco leaves co-infected with CsPAO2-YFP^n^ and PSA3-YFP^c^. Therefore, CsPAO2 interacted with PSA3 both in vivo and in vitro.

### 2.3. Subcellular Localization of Cucumber PSA3

**Figure 3:**
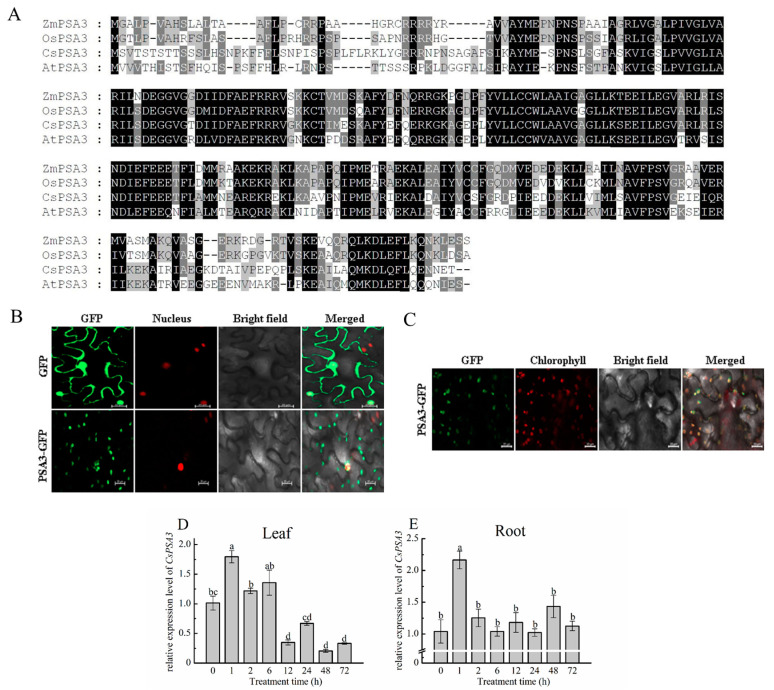
Amino acid sequence alignment (**A**) and subcellular localization (**B**,**C**) of CsPSA3, and time-course changes of *CsPSA3* expression in leaf (**D**) and root (**E**) of salt-treated cucumber. Cucumber (*Cucumis Sativus*, CsPSA3), Arabidopsis (*Arabidopsis thaliana*, AtPSA3), rice (*Oryza sativa*, OsPSA3), maize (*Zea mays*, ZmPSA3). The different letters indicate expression levels that were significant different between different treatment time (*p* < 0.05) according to Tukey’s test. Scale bars (20 μm) were added to the bottom right of each figure using ZEN 2 software.

Considering that so far there have been no reports about PSA3 in cucumber, the protein sequence was aligned with that from Arabidopsis, rice and maize firstly to further investigate the interaction between CsPAO2 and PSA3 and their function in response to salt stress. CsPSA3, OsPSA3, ZmPSA3 and AtPSA3 had conserved protein sequences in common, and the homology of amino acid sequence between cucumber and the other three plants was 57%, 53% and 59%, respectively (Figure 3A). Subcellular localization of CsPSA3 showed that the green fluorescence of PSA3-GFP was localized at cell membrane or cytoplasm as presented in spots and overlapped partly with nucleus (Figure 3B). Since *CsPSA3* encoded photosystem Ⅰ assembly 3, we speculated that CsPSA3 might also be localized in chloroplast such as PSA3 of other crops, which was confirmed by observing chloroplast auto-fluorescence (Figure 3C). Therefore, CsPSA3 is localized not only in the nucleus but also in the chloroplast, which is associated with the punctate green fluorescence in Figure 3B.

### 2.4. Expression Analysis of CsPSA3 under Salt Stress

PSA3 in maize and Arabidopsis was encoded by both the chloroplast and nuclear genomes, and they had different annotation, i.e., calcium homeostasis regulator [20,21]. Combining this fact with the localization of CsPSA3 in chloroplast and nucleus, it was speculated that CsPSA3 is also likely to be found in root besides leaf. Then, expression changes of *CsPSA3* under salt stress were determined in both leaf and root (Figure 3D,E). In leaf, the *CsPSA3* expression level was increased after 1h NaCl treatment and obviously decreased afterwards. Moreover, a greater decrease (75%) was found at 12 h when compared with 6 h and *CsPSA3* expression remained low after 12 h. Similarly, in root, the expression level of *CsPSA3* was increased by more than 1 fold from 0 h to 1 h and decreased to normal level at 2 h. However, no visible changes were observed after 2 h. Therefore, *CsPSA3*’s responses to salt stress in leaf and root were different, implying that *CsPSA3* might play different roles in root and leaf in response to salinity stress.

### 2.5. Effects of CsPAO2 and CsPSA3 Silencing on Photosynthesis of Cucumber under Salt Stress

**Figure 4:**
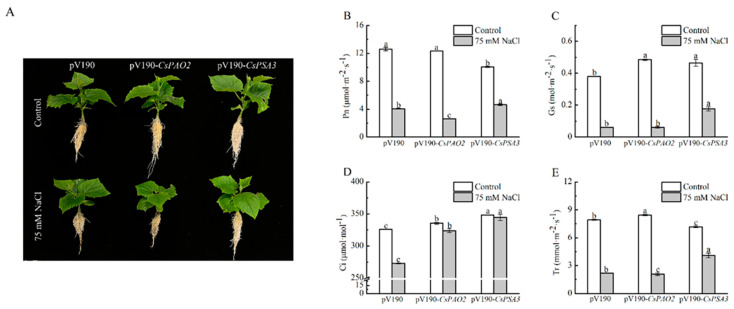
Effects of *CsPAO2* and *CsPSA3* silencing on photosynthesis of salt-treated cucumber. (**A**) Cucumber seedlings with or without NaCl treatment; (**B**) photosynthetic rate (Pn); (**C**) stomatal conductance (Gs); (**D**) intercellular CO_2_ (Ci); (**E**) transpiration rate (Tr). Gene-silencing plants cultivated in nutrient solution were treated with 75 mM NaCl for 3 days. The different letters indicate significantly different test index values between different gene-silencing cucumber plants under the same treatment (*p* < 0.05) according to Tukey’s test.

Gene encoding CsPAO2 or CsPSA3 was silenced by VIGS method in cucumber to further study the interaction between these two proteins and their role in salt stress. Because PSA3 was related to assembly of photosystem Ⅰ, the effects of gene silencing on photosynthesis were detected (Figure 4B–E). Under the control conditions, although *CsPAO2*-silencing cucumber plants displayed significantly higher Gs, Ci and Tr than pV190-silencing plants, the difference of Pn between these two plants was not significant. Gs and Ci of *CsPSA3*-silencing plants was both significantly higher than those of pV190-silencing plants, while the values of Tr and Pn were relatively lower. Compared with control conditions, except that Ci in *CsPAO2* and *CsPSA3*-silencing plants changed a little after NaCl treatment, the four photosynthetic parameters in all the tested plants were declined sharply, among which Pn and Tr of *CsPAO2*-silencing plants were significantly less than those of pV190-silencing plants and suffered more from limitation of salt stress on photosynthesis (Figure 4B,E). However, the four parameters of *CsPSA3*-silencing plants were all obviously higher than the other two silencing plants, indicating that downregulation of *CsPSA3* was beneficial to cucumber photosynthesis and growth under salt stress.

### 2.6. Effects of CsPAO2 and CsPSA3 Silencing on MDA and EL of Cucumber under Salt Stress

**Figure 5:**
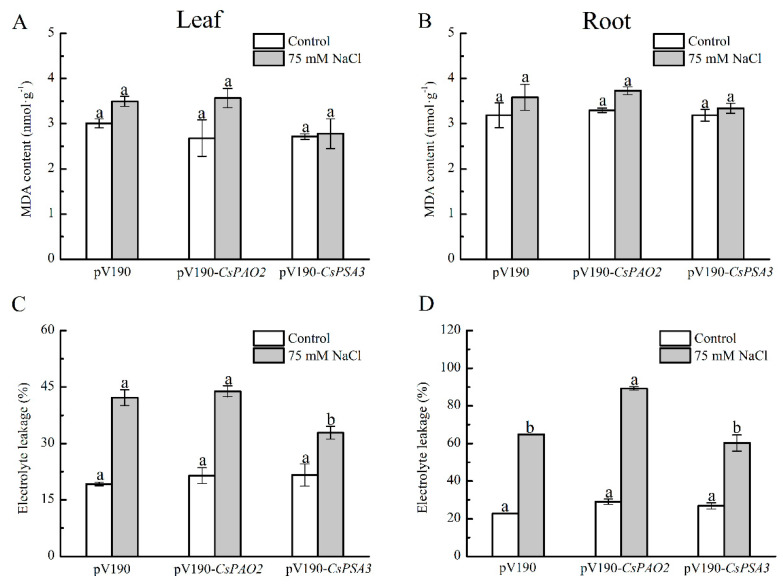
Effects of *CsPAO2* and *CsPSA3* silencing on MDA and EL of salt-treated cucumber. A, B, MDA content in leaf (**A**) and root (**B**). C, D, EL in leaf (**C**) and root (**D**). Gene-silencing plants cultivated in nutrient solution were treated with 75 mM NaCl for 3 days. The different letters indicate significantly different test index values between different gene-silencing cucumber plants under the same treatment (*p* < 0.05) according to Tukey’s test.

MDA and EL were measured to investigate the effects of *CsPAO2* and *CsPSA3* silencing on oxidative damage of cucumber caused by salt stress (Figure 5A–D). There was no significant difference of MDA content in leaf and root between the three silencing plants whether stressed or not, but it was noteworthy that the increasing range in *CsPAO2*-silencing plants treated with NaCl was larger relative to the other two silencing plants (33.2% in leaf, 13.0% in root) and the smallest one was found in *CsPSA3*-silencing plants (2.6% in leaf, 4.7% in root). Unlike MDA, EL was raised dramatically in the leaf and root of all tested plants exposed to salt stress, whereas the relative magnitude of EL change between different silencing plants was the same as MDA, and EL in root of *CsPAO2*-silencing plants was significantly higher than the other two plants (Figure 5D), suggesting that *CsPAO2*-silencing plants suffered most under salt stress. In salt-treated *CsPSA3*-silencing plants, EL of leaves was less than that of *CsPAO2*-silencing and pV190-silencing plants, while the difference of EL in root between *CsPSA3*-silencing and pV190-silencing plants was not significant (Figure 5C,D), indicating that there was less oxidative damage of *CsPSA3*-silencing plants resulting from salt stress. In addition, salt stress tolerance of *CsPSA3*-silencing plants was enhanced, particularly in leaves.

### 2.7. Effects of CsPAO2 and CsPSA3 Silencing on Polyamine Content of Cucumber under Salt Stress

**Figure 6:**
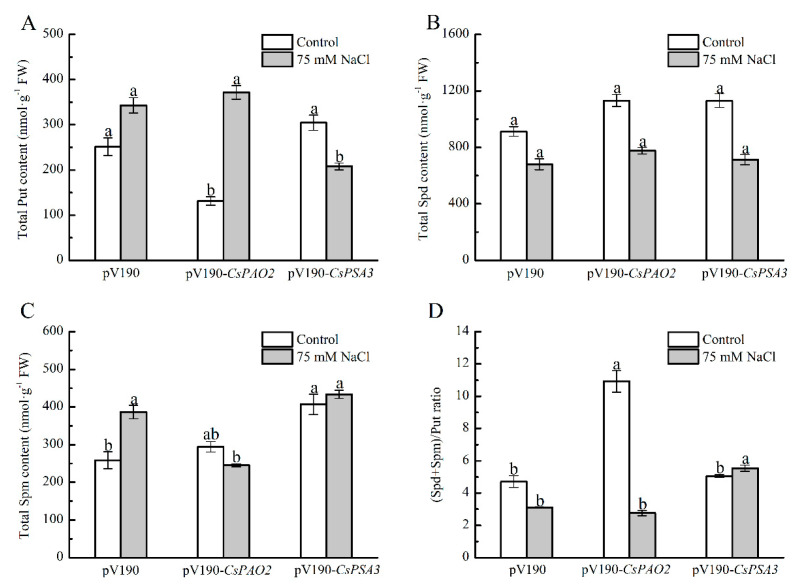
Effects of *CsPAO2* and *CsPSA3* silencing on polyamine content in leaf of salt-treated cucumber. (**A**) Put; (**B**) Spd; (**C**) Spm; (**D**) (Spd+Spm)/Put ratio. Gene-silencing plants cultivated in nutrient solution were treated with 75 mM NaCl for 3 days. The different letters indicate significantly different test index values between different gene-silencing cucumber plants under the same treatment (*p* < 0.05) according to Tukey’s test.

A significant alleviating effect of *CsPSA3* silencing on stress damage was observed in leaves (Figure 5), and then their effects on the polyamine content of leaf was investigated (Figure 6). Under the control conditions, Put level of *CsPAO2*-silencing plants was obviously lower than that of pV190-silencing plants, while Spd and Spm level was higher than pV190-silencing plants without significant difference, which was due to the inhibition of back-conversion of Spd and Spm caused by downregulation of *CsPAO2* expression and led to the markedly higher ratio of (Spd+Spm)/Put (Figure 6D). However, the contents of these three kinds of polyamines in *CsPSA3*-silencing plants were all higher than the other two gene-silencing plants, and it was speculated that *CsPSA3* silencing could induce the synthesis of polyamines. In addition, the lowest content of Spm and a sharp decrease in the (Spd+Spm)/Put ratio (3 fold) was observed in *CsPAO2*-silencing plants among the all salt-treated plants (Figure 6C,D), which might result from the less conversion from Put to Spd and Spm promoted by CsPAO2 function (Figure 1F–H), and this was likely to be recovered in *CsPSA3*-silencing plants whose (Spd+Spm)/Put ratio was insignificantly increased but significantly higher than the other gene-silencing plants under salt stress (Figure 6D).

## 3. Discussion

*Polyamine oxidases* (*PAOs*) are stress-responsive genes and play an important role in modulating the tolerance of plants to salt stress [10,11,12], which may vary in different members of the gene family. Therefore, our study started with the functional analysis of *CsPAO2* in salt stress resistance by overexpression in Arabidopsis plants. Similar to our previous result of *CsPAO3* [19], *CsPAO2* was also found to be a candidate gene for enhancing plants’ tolerance to salt, whether seen from the perspective of seed germination rate (Figure S3), oxidative damage (Figure 1B–D and Figure S5) or plant growth (Figure 1A and Figure S4). However, the difference between these two genes is that CsPAO2 functioned in the back conversion of Spd and Spm instead of terminal catabolism catalyzed by CsPAO3, due to which the (Spd+Spm)/Put ratio was reduced inevitably in *CsPAO2* overexpression plants (Figure 1H). A higher (Spd+Spm)/Put ratio would help plants resist abiotic stress [22,23], and thus preventing further decrease is vital for *CsPAO2* overexpression lines after salt stress. In our study, compared to control plants, this ratio in transgenic Arabidopsis plants was not decreased further, even increased in OE12 and OE18, indicating that increased CsPAO2 activity could promote the conversion from Put to Spd and Spm to keep the (Spd+Spm)/Put ratio from further decreases, despite the fact that they were in the opposite direction. On the other hand, due to downregulation of *CsPAO2* expression, *CsPAO2*-silencing plants were unable to maintain a high ratio of (Spd+Spm)/Put when exposed to salt stress and the ratio decreased dramatically (Figure 6), which provided further support to the conclusion described above.

Subsequently, CsPSA3 was discovered as a candidate interacting protein of CsPAO2 through yeast-two hybrid library screening and their interaction was confirmed by several methods (Figure 2), including in vivo and in vitro. *PSA3* has been characterized in several plants such as Arabidopsis, maize, rice and tomato. However, the interesting thing was that there were two different annotations for CsPSA3 in the cucumber genome database: photosystem Ⅰ (PSⅠ) assembly 3 and calcium homeostasis regulator 1, which were identical to maize, rice and Arabidopsis in each database and still remains unexplained [21]. There have been no studies of PSA in cucumber, but Yang, Liu, Wen and Lu [20] found that PSⅠ assembly protein was both encoded by both plastid and nuclear genes, and maize PSA3 was encoded by nuclear genes as annotated in NCBI, which was consistent with our observation of subcellular location for CsPSA3. It was localized in chloroplast and nucleus (Figure 3B,C). The expression profile of *CsPSA3* under salt stress showed that *CsPSA3* made responses differently both in leaf and root, suggesting that CsPSA3 might also play vital roles in root besides participation in assembly of PSⅠ in leaf. Moreover, there was a correlation between expression of *CsPSA3* and *CsPAO2* in response to salt stress when compared with our previous detection [19], i.e., their expression was both upregulated after 1 h of salt treatment in root, but in leaf, *CsPAO2* expression was enhanced at 6 h and expression level of *CsPSA3* was decreased dramatically at 12 h. So, we speculated that the interaction between these two proteins probably existed in both leaf and root.

In studies, polyamine was shown to bind with photosynthesis compounds and regulate secondary structures of light harvesting complex Ⅱ, PSⅠ and PSⅡ, thus affecting photosynthesis [24], and antisense-mediated *S-adenosyl-L-methionine decarboxylase* silencing which lacked synthesis of Spd and Spm, exhibited significantly reduced photosynthesis rate under heat stress [25]. In addition, the synthesis and degradation of polyamine was also regulated by light. The activity of ornithine decarboxylase, a key enzyme for Put synthesis, was increased rapidly in dark-treated *Chlamydomonas reinhardtii* cells after being transferred to light [26], and *OsPAO5* expression in rice was upregulated when exposed to light while it was inhibited under dark conditions [27]. A drought-tolerant maize genotype was previously found to have a higher PAO expression level and photosynthesis efficiency relative to the drought-sensitive genotype [13]. So, we supposed that CsPAO2 interacted with CsPSA3 to affect photosynthesis, whose change was measured after PAO2 and PSA3 were silenced in cucumber by VIGS. Under the control condition, photosynthesis was impacted because of the relationship between CsPSA3 and PSⅠ assembly, and Pn was reduced (Figure 4B). However, according to each photosynthetic parameter, photosynthesis of *CsPSA3*-silencing cucumber plants suffered significantly less damage than pV190-silecing plants after stress treatment. The increased level of *CsPSA3* expression at 1 h following salt treatment was probably for accelerating PSⅠ accumulation and enhancing its activity, and the decreased expression level at a later stage could induce an increase in Gs, Ci and Tr, which would in turn maintain the normal operation of photosynthesis under salt stress, illustrating that other roles might be played by CsPSA3 in photosynthesis. On the other hand, *CsPAO2* silencing caused more damage to photosynthesis, absorption and utilization of CO_2_ was declined and Ci was increased (Figure 4D).

Oxidative damage could be induced by salt stress, which leads to the increase in MDA content and EL. Similar to photosynthesis in leaf, the root of *CsPAO2*-silencing plants was more severely damaged in this study. However, we could not ignore the function of *CsPSA3*; EL in the leaf of *CsPSA3*-silencing plants was lower than that of the other two gene-silencing plants, while there was no marked difference between EL in the root of *CsPSA3* and pV190-silencing plants (Figure 5C,D), suggesting that *CsPSA3* played a more important role in leaf than in root and it was involved in the regulation of salt tolerance of leaf by *CsPAO2*. Then we found that CsPSA3 could also influence endogenous polyamine content whether under control or salt stress conditions, and notably, conversion from Put to Spd and Spm under salt stress was promoted by *CsPSA3* silencing, which was beneficial for plants to maintain a higher level of (Spd+Spm)/Put ratio and improve salt tolerance.

**Figure 7:**
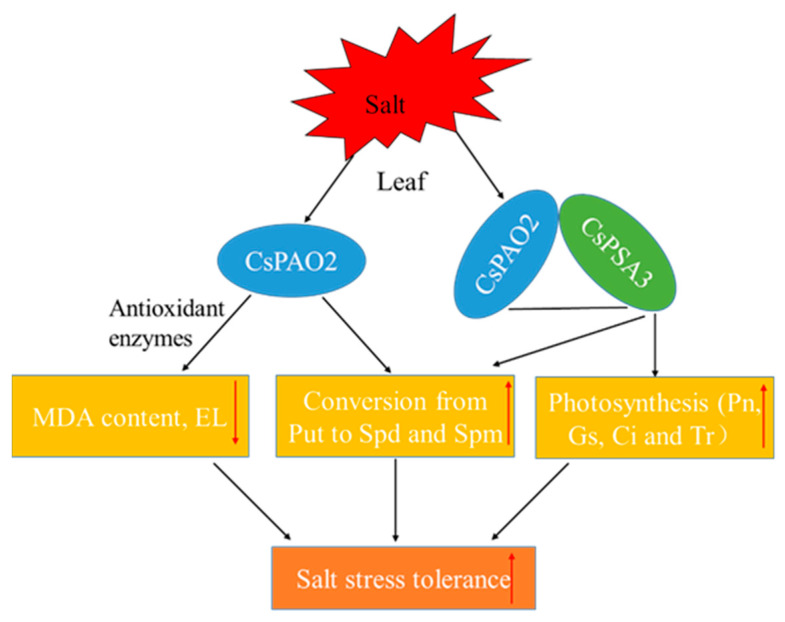
A proposed model of CsPAO2 enhancing salinity stress tolerance of cucumber through interaction with CsPSA3. Red arrows pointing up or down represent increase or decrease.

In summary, *CsPAO2* and *CsPSA3* responded to salt stress in both leaf and root, and they interacted to influence their regulation of salt stress tolerance. This interaction can be summarized as follows (Figure 7): *CsPAO2* expression was enhanced in leaf after salt stress, on the one hand, oxidative damage was alleviated by the increased activities of antioxidant enzyme induced by CsPAO2, and conversion between polyamines was also promoted; on the other hand, CsPAO2 interacted with CsPSA3 to elevate Gs, Ci and Tr, leading to improved photosynthesis, and the same conversion between polyamines was also accelerated, contributing to the enhanced salt tolerance. However, this regulatory mechanism needs further investigation, and how PSAS affects photosynthetic parameters, such as Gs and Ci, remains to be explored.

## 4. Materials and Methods

### 4.1. Plant Materials and Growth Conditions

Uniform seeds of cucumber (*Cucumis sativus* L.) were sown in quartz sand, and the seedlings were transferred to hydroponic cultivation in 1/2 Hoagland nutrient solution (pH 6.5 ± 0.1, electrical conductivity 2.0~2.2 mS·cm^−1^) at the second leaves stage. Cucumber seedlings were subjected to salt stress when the third leaves were fully expanded, i.e., the nutrient solution containing 75 mM NaCl. At 0, 1, 2, 6, 12, 24, 48 and 72 h after each treatment, the leaf and root samples were collected.

The floral dip method [28] was used to perform Arabidopsis (*Arabidopsis thaliana* L., cv. Columbia) transformation and homozygous T3 lines (OE7, OE12, OE18) were obtained, whose detailed procedure was described below in “Genetic transformation of Arabidopsis”. After disinfection, WT and transgenic Arabidopsis seeds were sown on a 1/2 Murashige-Skoog (MS) medium for 10 days, then seedlings of similar size were treated as follows: (1) seedlings were transferred to square plate containing 1/2 MS medium with various concentrations of NaCl (0, 100 and 200 mM) and cultivated vertically for 7 days to observe root growth; (2) seedlings were transplanted to vermiculite culture in square flower pots and they were watered with normal water or water supplemented with 200 mM NaCl for 10 days after 3-weeks of growth. All of the samples were frozen instantly in liquid nitrogen and kept at −80 °C for subsequent analysis.

Tobacco (*Nicotiana tabacum* L.) seeds were randomly sown in square flower plots, and seedlings were transferred to several plots (2 plants per plot) for subsequent agrobacteria infection.

### 4.2. Genetic Transformation of Arabidopsis

*CsPAO2* overexpression vector was constructed by homologous recombination method and introduced into Agrobacteria after sequencing confirmation. Arabidopsis seedlings were infected by this Agrobacteria according to the floral dip method [28]. Seeds of Arabidopsis plants after infection were collected and plated on a 1/2 MS medium containing kanamycin to screen positive plants growing normally (T1 generation), whose seeds were saved individually (15 plants in total). Seeds of each plant were then sown separately on a 1/2 MS medium supplemented with kanamycin, and lines showing 1:2:1/3:1 segregation ratio were selected (10 lines), among which green plants with relatively better growth were transferred to vermiculite cultivation (10 plants for each line) and seeds of each plant were collected (T2 generation). Subsequently, the seeds of each plant were sown respectively on a 1/2 MS medium containing kanamycin again to choose 5 plants with a relatively high growth uniformity from 10 plants and seeds were saved individually (T3 generation); the plant with higher quality of seeds was used as the representative plant of each line.

### 4.3. Arabidopsis Seed Germination Rate Analysis

A 1/2 MS medium supplemented with different concentration of NaCl (0, 100 and 200 mM) was used to sow seeds, and the number of geminated seeds was recorded once a day for 7 days. The germination rate was calculated by multiplying the proportion of germinated seeds by the total number of seeds sowed (%).

### 4.4. Estimation of Malondialdehyde (MDA) Content and Electrolyte Leakage (EL)

Samples were homogenized in trichloroacetic acid (TCA, 5%). After centrifugation at 4000× *g* for 10 min, 2 mL of supernatant was incorporated with thiobarbituric acid (TBA, 0.67%) of the equal volume, and the obtained mixture was centrifuged at 3000× *g* for 15 min after heated for 30 min using boiling water bath. The absorbance of the supernatant was measured at 450, 532 and 600 nm, which were used to calculate the MDA content according to the method of Dhindsa et al. [29]. The method described by Xu et al. [30] was used as a reference for EL detection with slight modifications. The samples were soaked with 20 mL of ddH_2_O in 50 mL tube, and kept at room temperature for 8 h and then conductivity was measured (initial conductivity, C_i_). After this, the tubes containing samples inside were autoclaved (121 °C, 20 min) and the conductivity (C_max_) was measured again. The proportion of C_i_ to C_max_ was calculated as EL (%).

### 4.5. Determination of H_2_O_2_ Content

The H_2_O_2_ content was measured according to the method developed by Alexieva et al. [31]. After grinding the samples in TCA (0.1%), the homogenates were centrifuged at 12,000× *g* for 20 min. 0.2 mL supernatant, 1 mL of 1 M KI solution and 0.25 mL of 0.1 M potassium phosphate buffer (pH 7.8) were mixed together and placed in darkness for 1 h. The absorbance of the mixture was read at 390 nm, and the H_2_O_2_ concentration was calculated with a standard curve based on serial concentrations gradient of H_2_O_2_ and the corresponding absorbance.

### 4.6. Antioxidant Enzyme Activity Assay

The enzymatic extract was obtained by using pre-cooled 0.05 M phosphate buffer (pH 7.8) to grind the samples and centrifuged at 12,000× *g* for 20 min, then the supernatant was used to measure enzymes activity. The nitro blue tetrazolium (NBT) photochemical reduction method was used to detect superoxide dismutase (SOD) activity [32]. One unit of SOD activity was defined as the amount of enzyme required to inhibit 50% of NBT reduction. Peroxidase (POD) and catalase (CAT) activities were measured using the method of Lin and Kao [33] and Li et al. [34], respectively. The reaction solution for POD activity assay included 0.2 M phosphate buffer (PBS, pH 6.0), 3.5 M guaiacol, and 30% H_2_O_2_, while that the solution for CAT assay included 30% H_2_O_2_ and PBS with different concentration and pH (0.05 M, pH 7.0). One unit of POD or CAT activity was defined as an increase of 0.01 A_470_·or a decrease of A_240_ per minute. For the measurement of ascorbate peroxidase (APX) activity, the enzyme extract was mixed with reaction solution containing 0.05 M PBS (pH 7.0) containing 0.1 mM EDTA-Na_2_, 5 mM ascorbic acid and 20 mM H_2_O_2_; its activity was then calculated by the method described by Nakano and Asad [35] using the decrease in A_290_ within 1 min.

### 4.7. Measurement of Polyamines Content

The polyamine content was determined according to the method of Shu et al. [36]. Leaf sample was ground in 1.6 mL of pre-cooled perchloric acid (PCA, 5%) followed by 20 min centrifugation at 12,000× *g* and 4 °C. The supernatant was collected for detecting free and conjugated Pas, while the pellet was used for the assay of bound Pas. 1.4 mL of 2 M NaOH and 15 μL benzoyl chloride was added to 0.7 mL supernatant and then placed at 30 °C for 30 min after 20-s vortex. Next, 2 mL of saturated NaCl solution and cold diethyl ether was mixed with the above solution and centrifuged at 12,000× *g*, 4 °C for 5 min, 1 mL of ether phase on the top was placed in new tubes until evaporation to dryness and 1 mL of chromatographically pure methanol was used to redissolve benzoyl Pas. Conjugated and bound Pas were extracted after 18-h hydrolyzation at 110 °C in sealed ampoule bottle. The samples redissolved in methanol were stored at −20 °C and filtered before testing. A UPLC system (Thermo, UltiMate 3000, Waltham, MA, USA) was used for detection the content of Pas.

### 4.8. Quantitative Real-Time PCR Analysis

RNAsimple Total RNA Kit (TIANGEN, China) was used to extract total RNA according to the manufacturer’s instructions. Then cDNA was synthesized through reverse transcription of total RNA (1 μg) using HiScript^®^ Ⅲ RT SuperMix for qPCR (Vazyme, Nanjing, China). Beacon Designer 7.9 (Premier Biosoft International, CA, USA) was used to design primers and presented in Table S1. QuantStudio^TM^ 6 Real-Time PCR System (Applied Biosystems) with ChamQ SYBR qPCR Master Mix (Vazyme, China) was applied for qRT-PCR. The relative expression of the gene was calculated by the 2^−ΔΔCT^ method [37] with the *actin* gene of cucumber or Arabidopsis as the internal reference gene.

### 4.9. Amino Acid Sequence Alignment of PSA3

The amino acid sequence of PSA3 was aligned by ClustalX (version 1.81) and Genedoc (version 2.7) software.

### 4.10. Observation of PSA3 Localization at Subcellular Level

The coding sequence (CDS) of *PSA3* without stop codon was amplified using primers pAC402-PSA3_F/R_ (Supplementary Table S1) and then inserted into the N terminal of the GFP gene in the pAC402 vector. The empty vector and the constructed vector was transformed into Agrobacteria after sequence confirmation, which were then transiently transformed into tobacco leaves through the syringe infiltration method [38]. After 12 h of exposure to dark treatment, tobacco plants were grown under normal conditions for 2 days, and tobacco leaves were observed and images were obtained using confocal laser scanning microscope (LSM 780, Zeiss, Oberkochen, Germany).

### 4.11. Determination of Photosynthetic Parameters

The LI-6400 portable photosynthesis system (LI-COR Inc., Lincoln, NE, USA) was used to detect Pn, intercellular CO_2_ concentration (Ci), stomatal conductance (Gs) and Tr values with light intensity, chamber temperature, relative humidity and CO_2_ concentration set as 1000 μmol·m^−2^·s^−1^, 25 °C, 70% and 400 ± 10 μmol·mol^−1^, respectively, were maintained.

### 4.12. Yeast Two-Hybrid cDNA Library Screening and Assay

The CDS of *CsPAO2* was ligated into the bait vector pGBKT7, and the yeast two-hybrid cDNA library of cucumber constructed by Meiwen [39] was screened with the yeast cotransformation method. The positive clones were sequenced and analyzed by sequence blast. Next, yeast two-hybrid validation of interaction between proteins was carried out. PSA3 were fused to the pGADT7 vector as prey plasmids and co-transformed into the yeast strain Y2H Gold with the bait plasmids, among which yeast cotransformed with pGBKT7-53 and pGADT7-T was used as a positive control, while the one cotransformed with pGBKT7-Lam and pGADT7-T was used as a negative control, and then transformed yeast was plated on SD-Trp/-Leu, SD-Trp/-Leu/-His and SD-Trp/-Leu/-His/-Ade plates.

### 4.13. Bimolecular Fluorescence Complementation (BiFC) Analysis

The full-length CDSs (without stop codon) of *CsPAO2* and *PSA3* were inserted separately into the ZYN and ZYC vectors. The methods of vector construction, Agrobacteria transformation and tobacco infection were the same as described above except that Agrobacteria carrying different recombinant plasmids should be mixed in equal volumes according to the protein combination needed to verify the interaction before injection. Confocal laser scanning microscope (LSM 780, Zeiss, Germany) was used to observe YFP fluorescence of tobacco leaves after two-days of growth.

### 4.14. Luciferase Complementation Assay (LCA)

LCA was conducted by the method of Hou et al. [40]. In accordance with the method of vector construction and Agrobacteria transformation in subcellular localization of PSA3, the CDSs of CsPAO2 and PSA3 were cloned into the nLUC and cLUC vectors, respectively, and then transformed into agrobacteria. Similar to the BiFC assay, Agrobacteria must be mixed prior to injection, and the difference is that the injection area was a circle rather than the whole leaf. After 2 days of growth, D-Luciferin potassium salt was evenly sprayed on the leaves as substrate and plant living imaging system (Lumazone PLXIS 1024B, Princeton Instruments, Trenton, NJ, USA) was used to observe luminescence.

### 4.15. GST Pull Down Assay

The method of vector construction mentioned above was used to ligate the CDS of *CsPAO2* into pET32a and the CDS of *PSA3* into pGEX4T-1. The constructed vector was then transformed into *Escherichia coli* Rosseta (DE3) to perform prokaryotic expression and extract protein. The solubilities of recombinant proteins and optimum induction time were tested first: incubated overnight grown culture in 20 mL of LB broth at 37 °C until OD_600_ achieved 0.6–0.8; 500 μL of culture was taken out and the pellets were collected at -80 °C for further analysis after centrifugation at 5000× *g*, 4 °C for 15 min; IPTG was added to the remanent culture and continued to grow at 25 °C with shaking; 500 μL of the culture was taken out at 2, 6, 8, 10 and 12 h after addition of IPTG, and the pellet was collected and stored at the same time; 100 μL of BugBuster master mix (Novagen) was used to resuspend collected pellet and 20 μL of solution was used as total protein after placed at room temperature for 15 min, then the rest was centrifuged, and the supernatant was used as soluble protein; at last, SDS-PAGE was performed after protein denaturation.

Next, inoculate the culture overnight into the larger volume of LB (400 mL) to extract enough protein according to the optimal induction time learned from the previous step. The pellet was collected after centrifugation and resuspended in 1 × PBS, and high- pressure cell cracker (JNBIO, Guangzhou, China) was used to break cells until the suspension became clear. Then, the supernatant was collected and stored at −80 °C after centrifugation. For pGEX4T-1-PSA3, a recombinant protein with low solubility, its protein was contained in the pellet and needed to be extracted after dialysis treatment.

GST-tag Purification Resin (BeyoGold, Shanghai, China) was used to perform GST pull down with the manufacturer instructions. The samples were separated by SDS-PAGE after denaturation and analyzed by Western blot with anti-His or anti-GST antibodies.

### 4.16. Co-Immunoprecipitation (Co-IP) Assay

The CDSs of *CsPAO2* and *PSA3* were ligated into pAC402 carrying GFP tag and pAC330 carrying Myc tag, respectively. The method of vector construction and tobacco injection was the same as described above. A total of 1 g samples of infected tobacco leaf were used for protein extraction: leaves powder was obtained after leaves ground in liquid nitrogen and extraction buffer (50 mM Tris-HCl (pH 8.0), 150 mM NaCl, 1 mM EDTA, 1% NP-40, 1% sodium deoxycholate, 0.1% SDS, 1 mM PMSF) was added to produce the homogenate and the supernatant was collected as protein extract after centrifugation. Then Anti-c-Myc magnetic beads (MedChemExpress, South Brunswick, NJ, USA) was used to conduct Co-IP assay following the instructions. Samples were analyzed via Western blot.

### 4.17. VIGS in Cucumber Plants

The vector pV190 used for VIGS in cucumber was provided by the lab of Gu et al. [41]. The methods of vector construction and Agrobacteria transformation were the same as mentioned above; meanwhile, as the control, the empty vector and the vector silencing *phytoene desaturase* (*PDS*) were also transformed into agrobacteria. Germinated-seed vacuum inoculation system was used to inject cucumber. Cucumber seeds were treated with bud forcing and vacuum infiltrated twice in infection solution for 10 min when the length of radicle reached about 5 mm. After injection, seeds were sown in substrate. Then, leaves were sampled for RNA extraction and qRT-PCR analysis of *CsPAO2* and *PSA3* expression until the leaves of cucumber plants infected by agrobacteria carrying *PDS*-silencing plasmid showed photobleaching symptoms (Figure S1), and the plants whose *CsPAO2* or *PSA3* expression level was lower than 0.5 were transferred to a hydroponic culture and treated with 75 mM NaCl. About 120 sprouted seeds were used for the vacuum infiltration of each gene every time, and 96 of which with uniform size were sown after injection. The efficiency of gene silencing was around 30% to 50%.

### 4.18. Statistical Analysis

There were at least three independently tested biological replicates for each experiment, and the values are the means ± SE of these independent experiments. The bars represent the standard error. All the data were statistically analyzed with the SPSS 17.0 software program (SPSS Inc., Chicago, IL, USA) and differences between treatments were compared using Tukey’s test at the *p* < 0.05 level of significance.

## Data Availability

The data sources compiled and analyzed during the present research are available from the corresponding author upon reasonable request.

## Supplementary Materials

The following supporting information can be downloaded at: https://www.mdpi.com/article/10.3390/ijms232012413/s1.

## References

[1] Alcazar R., Altabella T., Marco F., Bortolotti C., Reymond M., Koncz C., Carrasco P., Tiburcio A.F. (2010). Polyamines: Molecules with regulatory functions in plant abiotic stress tolerance. Planta.

[2] Pal M., Szalai G., Janda T. (2015). Speculation: Polyamines are important in abiotic stress signaling. Plant Sci..

[3] Kim D.W., Watanabe K., Murayama C., Izawa S., Niitsu M., Michael A.J., Berberich T., Kusano T. (2014). Polyamine Oxidase5 Regulates Arabidopsis Growth through Thermospermine Oxidase Activity. Plant Physiol..

[4] Alabdallah O., Ahou A., Mancuso N., Pompili V., Macone A., Pashkoulov D., Stano P., Cona A., Angelini R., Tavladoraki P. (2017). The Arabidopsis polyamine oxidase/dehydrogenase 5 interferes with cytokinin and auxin signaling pathways to control xylem differentiation. J. Exp. Bot..

[5] Tavladoraki P., Cona A., Angelini R. (2016). Copper-Containing Amine Oxidases and FAD-Dependent Polyamine Oxidases Are Key Players in Plant Tissue Differentiation and Organ Development. Front. Plant Sci..

[6] Cona A., Rea G., Angelini R., Federico R., Tavladoraki P. (2006). Functions of amine oxidases in plant development and defence. Trends Plant Sci..

[7] Wang W., Zheng X., Liu S., Tan B., Feng J. (2021). Polyamine oxidase (PAO)–mediated polyamine catabolism plays potential roles in peach (*Prunus persica* L.) fruit development and ripening. Tree Genet. Genomes.

[8] Wu J., Shang Z., Wu J., Jiang X., Moschou P.N., Sun W., Roubelakis-Angelakis K.A., Zhang S. (2010). Spermidine oxidase-derived H2O2 regulates pollen plasma membrane hyperpolarization-activated Ca2+-permeable channels and pollen tube growth. Plant J..

[9] Rodríguez A.A., Maiale S.J., Menéndez A.B., Ruiz O.A. (2009). Polyamine oxidase activity contributes to sustain maize leaf elongation under saline stress. J. Exp. Bot..

[10] Moschou P.N., Paschalidis K.A., Delis I.D., Andriopoulou A.H., Lagiotis G.D., Yakoumakis D.I., Roubelakis-Angelakis K.A. (2008). Spermidine Exodus and Oxidation in the Apoplast Induced by Abiotic Stress Is Responsible for H2O2 Signatures That Direct Tolerance Responses in Tobacco. Plant Cell.

[11] Moschou P.N., Sanmartin M., Andriopoulou A.H., Rojo E., Sanchez-Serrano J.J., Roubelakis-Angelakis K.A. (2008). Bridging the Gap between Plant and Mammalian Polyamine Catabolism: A Novel Peroxisomal Polyamine Oxidase Responsible for a Full Back-Conversion Pathway in Arabidopsis. Plant Physiol..

[12] Xiong H., Guo H., Xie Y., Zhao L., Gu J., Zhao S., Li J., Liu L. (2017). RNAseq analysis reveals pathways and candidate genes associated with salinity tolerance in a spaceflight-induced wheat mutant. Sci. Rep..

[13] Pakdel H., Hassani S.B., Ghotbi-Ravandi A.A., Bernard F. (2020). Contrasting the expression pattern change of polyamine oxidase genes and photosynthetic efficiency of maize (Zea mays L.) genotypes under drought stress. J. Biosci..

[14] Hatmi S., Trotel-Aziz P., Villaume S., Couderchet M., Clément C., Aziz A. (2013). Osmotic stress-induced polyamine oxidation mediates defence responses and reduces stress-enhanced grapevine susceptibility to Botrytis cinerea. J. Exp. Bot..

[15] Gémes K., Mellidou Ι., Karamanoli K., Beris D., Park K.Y., Matsi T., Haralampidis K., Constantinidou H.-I., Roubelakis-Angelakis K.A. (2017). Deregulation of apoplastic polyamine oxidase affects development and salt response of tobacco plants. J. Plant Physiol..

[16] Wang W., Liu J.-H. (2016). CsPAO4 of Citrus sinensis functions in polyamine terminal catabolism and inhibits plant growth under salt stress. Sci. Rep..

[17] Gémes K., Kim Y.J., Park K.Y., Moschou P.N., Andronis E., Valassaki C., Roussis A., Roubelakis-Angelakis K.A. (2016). An NADPH-Oxidase/Polyamine Oxidase Feedback Loop Controls Oxidative Burst Under Salinity. Plant Physiol..

[18] Toumi I., Pagoulatou M.G., Margaritopoulou T., Milioni D., Roubelakis-Angelakis K.A. (2019). Genetically Modified Heat Shock Protein90s and Polyamine Oxidases in Arabidopsis Reveal Their Interaction under Heat Stress Affecting Polyamine Acetylation, Oxidation and Homeostasis of Reactive Oxygen Species. Plants.

[19] Wu J., Liu W., Jahan M.S., Shu S., Sun J., Guo S. (2021). Characterization of polyamine oxidase genes in cucumber and roles of CsPAO3 in response to salt stress. Environ. Exp. Bot..

[20] Yang H., Liu J., Wen X., Lu C. (2015). Molecular mechanism of photosystem I assembly in oxygenic organisms. Biochim. Biophys. Acta.

[21] Shen J., Williams-Carrier R., Barkan A. (2017). PSA3, a Protein on the Stromal Face of the Thylakoid Membrane, Promotes Photosys tem I Accumulation in Cooperation with the Assembly Factor PYG7. Plant Physiol..

[22] Bouchereau A., Aziz A., Larher F., Martin-Tanguy J. (1999). Polyamines and environmental challenges: Recent development. Plant Sci..

[23] Gupta K., Dey A., Gupta B. (2013). Plant polyamines in abiotic stress responses. Acta Physiol. Plant..

[24] Pál M., Szalai G., Gondor O.K., Janda T. (2021). Unfinished story of polyamines: Role of conjugation, transport and light-related regulation in the polyamine metabolism in plants. Plant Sci..

[25] Mellidou I., Karamanoli K., Constantinidou H.-I.A., Roubelakis-Angelakis K.A. (2020). Antisense-mediated S-adenosyl-L-methionine decarboxylase silencing affects heat stress responses of tobacco plants. Funct. Plant Biol..

[26] Voigt J., Deinert B., Bohley P. (2000). Subcellular localization and light-dark control of ornithine decarboxylase in the unicellular green alga Chlamydomonas reinhardtii. Physiol. Plant..

[27] Lv Y., Shao G., Jiao G., Sheng Z., Xie L., Hu S., Tang S., Wei X., Hu P. (2021). Targeted mutagenesis of POLYAMINE OXIDASE 5 that negatively regulates mesocotyl elongation enables the generation of direct-seeding rice with improved grain yield. Mol. Plant.

[28] Zhang X., Henriques R., Lin S.-S., Niu Q.-W., Chua N.-H. (2006). Agrobacterium-mediated transformation of Arabidopsis thaliana using the floral dip method. Nat. Protoc..

[29] Dhindsa R.S., Plumb-Dhindsa P., Thorpe T.A. (1981). Leaf Senescence: Correlated with Increased Levels of Membrane Permeability and Lipid Peroxidation, and Decreased Levels of Superoxide Dismutase and Catalase. J. Exp. Bot..

[30] Xu Y., Burgess P., Zhang X., Huang B. (2016). Enhancing cytokinin synthesis by overexpressing ipt alleviated drought inhibition of root growth through activating ROS-scavenging systems in Agrostis stolonifera. J. Exp. Bot..

[31] Alexieva V., Sergiev I., Mapelli S., Karanov E. (2001). The effect of drought and ultraviolet radiation on growth and stress markers in pea and wheat. Plant Cell Environ..

[32] Becana M., Aparicio-Tejo P., Irigoyen J.J., Sanchez-Diaz M. (1986). Some Enzymes of Hydrogen Peroxide Metabolism in Leaves and Root Nodules of *Medicago sativa*. Plant Physiol..

[33] Lin C.C., Kao C.H. (2001). Abscisic acid induced changes in cell wall peroxidase activity and hydrogen peroxide level in roots of rice seedlings. Plant Sci..

[34] Li Z., Zhang Y., Peng D., Wang X., Peng Y., He X., Zhang X., Ma X., Huang L., Yan Y. (2015). Polyamine regulates tolerance to water stress in leaves of white clover associated with antioxidant defense and dehydrin genes via involvement in calcium messenger system and hydrogen peroxide signaling. Front. Physiol..

[35] Nakano Y., Asad K. (1980). Hydrogen Peroxide is Scavenged by Ascorbate-specific Peroxidase in Spinach Chloroplasts. Plant Cell Physiol..

[36] Shu S., Guo S.R., Sun J., Yuan L.Y. (2012). Effects of salt stress on the structure and function of the photosynthetic apparatus in Cucumis sativus and its protection by exogenous putrescine. Physiol. Plant.

[37] Livak K.J., Schmittgen T.D. (2001). Analysis of relative gene expression data using real-time quantitative PCR and the 2(-Delta Delta C(T)) Method. Methods.

[38] Schweiger R., Schwenkert S. (2014). Protein-protein Interactions Visualized by Bimolecular Fluorescence Complementation in Tobacco Protoplasts and Leaves. J. Vis. Exp..

[39] Meiwen H. (2019). Identification of S-adenosylmethionine Synthetase Gene and Its Salt Stress Response Function in Cucumber. Ph.D. Thesis.

[40] Hou K., Wang Y., Tao M.-Q., Jahan M.S., Shu S., Sun J., Guo S.-R. (2020). Characterization of the CsPNG1 gene from cucumber and its function in response to salinity stress. Plant Physiol. Biochem..

[41] Liu M., Liang Z., Aranda M.A., Hong N., Liu L., Kang B., Gu Q. (2020). A cucumber green mottle mosaic virus vector for virus-induced gene silencing in cucurbit plants. Plant Methods.

