# Supplementary material for: CsPAO2 Improves Salt Tolerance of Cucumber through the Interaction with CsPSA3 by Affecting Photosynthesis and Polyamine Conversion
Source: Int J Mol Sci. 2022 Oct 17;23(20):12413. doi: 10.3390/ijms232012413 (PMC9604536; doi:10.3390/ijms232012413)
Supplement: Supplementary file 1 [file ijms-23-12413-s001.zip › ijms-1944586-supplementary.pdf]

Table S1. Primers used in this study.

Primer name	Forward primer (5’ to 3’)	Reverse primer (5’ to 3’)
AtActin	GGTAACATTGTGCTCAGTGGTGG	AACGACCTTAATCTTCATGCTGC
GFP	CATCTTCTTCAAGGACGA	GTGGCTGTTGTAGTTGTA
pAC402-PSA3	tacaaatctatctctctcgagATGTCCGTGACCTCAACTAGCAC	GgatccccgggtaccgagctcAGTTTCATTATTCTCTTGGAGAAATTGG
pAC013-PAO2	ggactctagaggatccccgggATGGAATCCGGAGCCAAGAGTAATTCCGA	gcccttgctcaccatggtacc CATTCGAGAAATTAACAAAGGTGCCGATA
BD-PAO2	atggccatggaggccgaattcATGGAATCCGGAGCCAAGA	GcaggtcgacggatccccgggCATTCGAGAAATTAACAAAGGTGC
AD-PSA3	gccatggaggccagtgaattcATGTCCGTGACCTCAACTAGCAC	cagctcgagctcgatggatccTTAAGTTTCATTATTCTCTTGGAGAAATT
ZYN-PAO2	atttacgaacgatagttaattaaATGGAATCCGGAGCCAAGA	actgccacctcctccactagtCATTCGAGAAATTAACAAAGGTGC
ZYC-PSA3	atttacgaacgatagttaattaaATGTCCGTGACCTCAACTAGCAC	actgccacctcctccactagtAGTTTCATTATTCTCTTGGAGAAATTGG
nLUC-PAO2	acgggggacgagctcggtaccATGGAATCCGGAGCCAAGA	cagtcgacgcgttgtggatccCATTCGAGAAATTAACAAAGGTGC
cLUC-PSA3	tacgcgtcccggggcggtaccATGTCCGTGACCTCAACTAGCAC	cagtcgacgcgttgtggatccTTAAGTTTCATTATTCTCTTGGAGAAATT
pET32a-PAO2	catggctgatatcggatccgaattcATGGAATCCGGAGCCAAGAGTAATTCCGA	agtgcggccgcaagctttgtcgacTTACATTCGAGAAATTAACAAAGGTGCCG
pGEX4T1-PSA3	aatctggttccgcgtggatccATGTCCGTGACCTCAACTAGCAC	CtcgagtcgacccgggaattcTTAAGTTTCATTATTCTCTTGGAGAAATT
pAC330-PSA3	catatggggctgcaggaattcATGTCCGTGACCTCAACTAGCAC	gggactagaactagtggatccTTAAGTTTCATTATTCTCTTGGAGAAATT
pV190-PAO2	aggactttacttaatggatccTATCGTTCGTGGTGGGGTGT	cctagacctataactggatccCAAACTTAGGTAATAGTTTTAGATCCATAAAA
pV190-PSA3	aggactttacttaatggatccGAGTCTAAAGCTTTCTATGAATTTCAAGA	cctagacctataactggatccAGCCTCATTCATCATAGCAAGGA

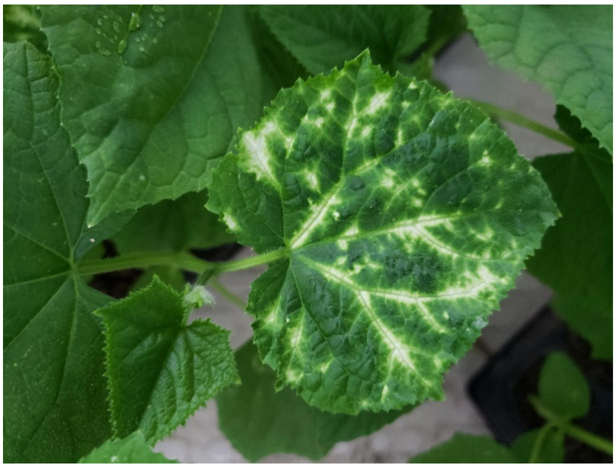

Figure S1. White streaks characteristic of silencing PDS in cucumber.

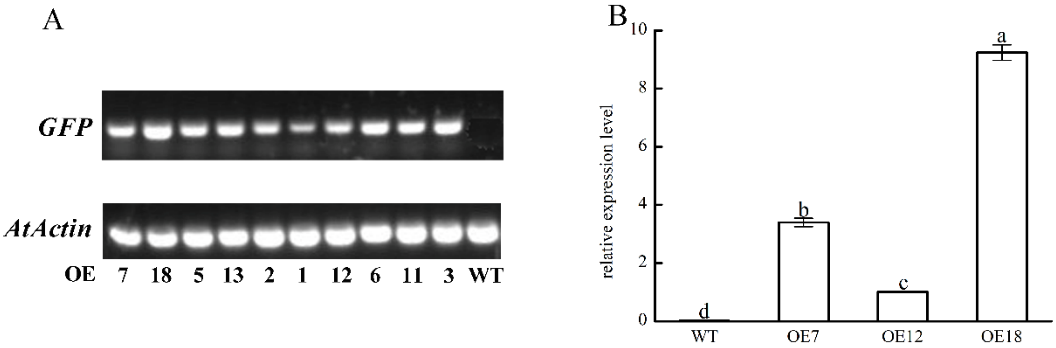

Figure S2. Semi-quantitative PCR analysis of *CsPAO2* expression and relative expression level of *CsPAO2* in OE7, OE12 and OE18. A, semi-quantitative PCR analysis; B, relative expression level of *CsPAO2* in OE7, OE12 and OE18. Relative expression level of *CsPAO2* in OE12 was taken as 1.

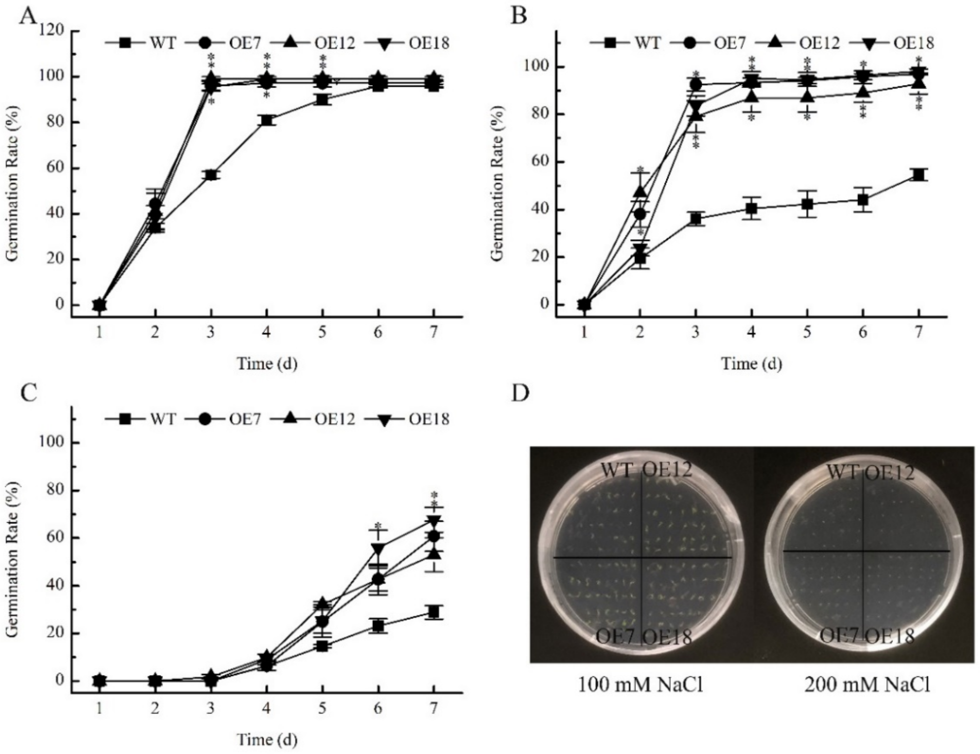

Figure S3. Seed germination rate of wild type (WT) and transgenic Arabidopsis lines (OE7, OE12 and OE18). A-C, time-course change of germination rate under 0 (A), 100 (B) and 200 (C) mM NaCl treatment. D, pictures of seeds grown on plates containing 100 and 200 mM NaCl for 7 days. Error bars represent standard error of three plates. Asterisks above lines indicate significant differences between WT and transgenic lines on the same day at $P < 0.05$ (*) according to Tukey test.

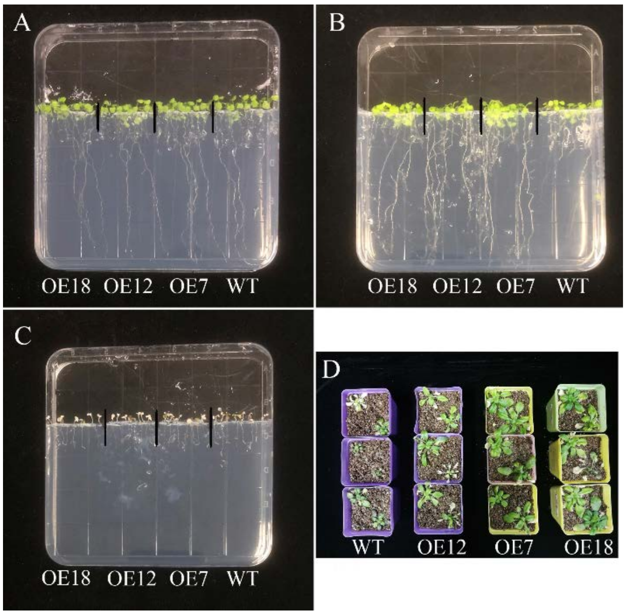

Figure S4. Root growth of wild type (WT) and transgenic plants (OE7, OE12 and OE18) under salt

stress. A-C, after 7 days of sowing on 1/2 MS medium without NaCl , seedlings were transferred onto 1/2 MS medium containing 0 (A), 100 (B) and 200 (C) mM NaCl, respectively, and pictures were taken on the seventh day. D, phenotype of Arabidopsis seedlings treated with 300 mM NaCl.

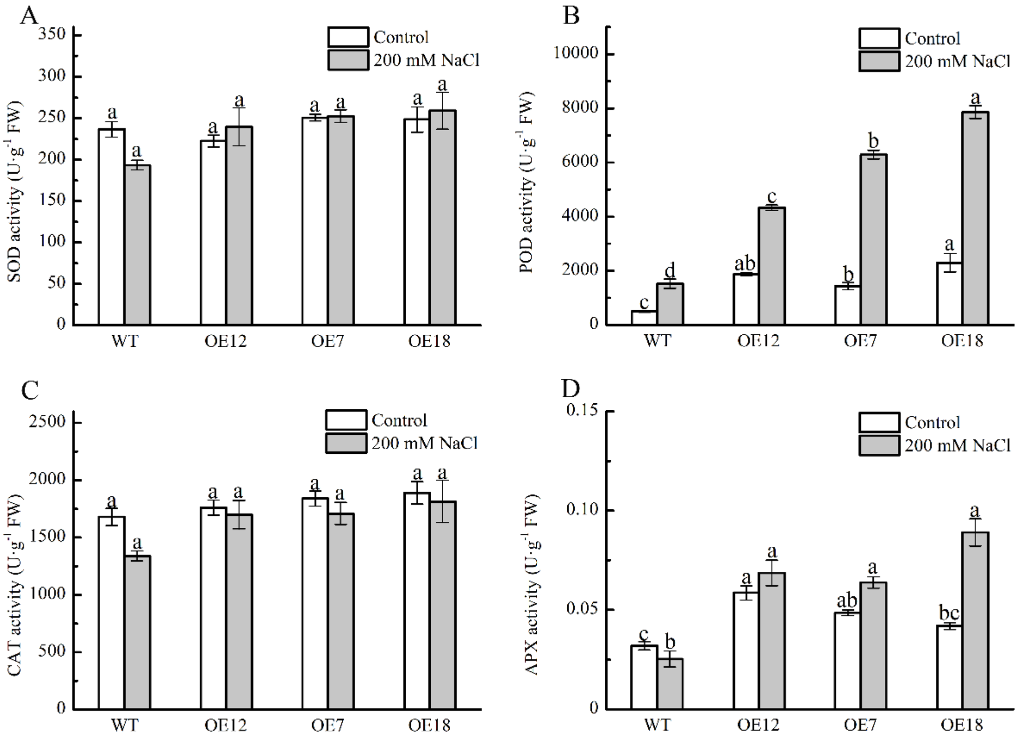

Figure S5. Antioxidant enzyme activities of wild type (WT) and transgenic Arabidopsis lines (OE12, OE7 and OE18). A, SOD; B, POD; C, CAT; D, APX. Arabidopsis seedlings cultivated in vermiculite were irrigated with 200 mM NaCl solution or water for 14 days. Error bars represent standard error of three replicates. The different letters indicate significant differences at $P < 0.05$ between different genotypes under the same treatment according to Tukey test.
